# Predictive Factors for the Discontinuation of Renal Replacement Therapy in Critically Ill Adults: A Systematic Review and Meta-Analysis

**DOI:** 10.7759/cureus.81783

**Published:** 2025-04-06

**Authors:** Eman Nasr Taha, Alaa H Ewida, Nehal N Elsheshtawi, Shimaa A Ragab, Dina Alaraby, Rasha Ewida, Ahmed Elmalky, Mohamad-Hani Temsah, Sidharth K Sethi, Rupesh Raina, Khalid Alhasan

**Affiliations:** 1 Central Administration of Drug Control, Egyptian Drug Authority, Cairo, EGY; 2 Medicine, Suez Canal University, Suez, EGY; 3 Research and Development, Clinmax Contract Research Organization (CRO), Cairo, EGY; 4 Research and Development, Medical Agency for Research and Statistics, Giza, EGY; 5 Radiology, Ain Shams University, Cairo, EGY; 6 Public Health, Theodore Bilharz Research Institute, Giza, EGY; 7 Morbidity and Mortality, King Saud University Medical City, Riyadh, SAU; 8 Pediatric Intensive Care Unit, Pediatrics, King Saud University Medical City, Riyadh, SAU; 9 Evidence-Based Research Chair, Department of Family Medicine, King Saud University, Riyadh, SAU; 10 Pediatric Nephrology, Kidney Institute, Medanta-The Medicity, Gurgaon, IND; 11 Nephrology, Akron Nephrology Associates/Cleveland Clinic Akron General Medical Center, Akron Children’s Hospital. Rebecca D. Considine Research Institute, Akron, USA; 12 Pediatric Nephrology, King Saud University, Riyadh, SAU; 13 Kidney and Pancreas Health Center, Organ Transplant Center of Excellence, King Faisal Specialist Hospital & Research Center, Riyadh, SAU; 14 Nephrology, Saudi Society of Nephrology and Transplantation, Riyadh, SAU

**Keywords:** acute kidney injury, continuous renal replacement therapy (crrt), icu los, length of hospital stay (los), medical icu, neutrophil gelatinase-associated lipocalin (ngal), predictive factors for crrt weaning, renal replacement therapy, urinary creatinine, urine output

## Abstract

Acute kidney injury (AKI) is a decline in kidney function. Acute kidney injury frequently occurs as a complication among patients who are hospitalized or critically ill. Consequently, we aimed to examine the factors that could predict the cessation of renal replacement therapy (RRT) in individuals with severe AKI. We conducted a systematic review and meta-analysis with a comprehensive literature search in PubMed, Excerpta Medica database (Embase), and the Cochrane Library to identify relevant studies exploring factors associated with a successful transition from continuous renal replacement therapy (CRRT). The search was conducted from each database from beginning until December 1, 2022. The research was carried out on adult critically ill patients taking RRT while being supported in an intensive care unit (ICU) environment.We identified a total of 11 studies through our search. The pooled analysis demonstrated several predictive factors for successful weaning from CRRT, including CRRT duration, urine output in the course of CRRT termination (with an increase of 100 mL/day), creatinine clearance, urinary creatinine (UCr), neutrophil gelatinase-associated lipocalin (NGAL), and ICU length of stay (p < 0.00001). We concluded that the correlation between predictive factors and the weaning process from CRRT is not yet well understood. Furthermore, evidence is scarce concerning the optimal threshold values for UO and serum creatinine levels to determine successful weaning from CRRT. These findings provide valuable guidance to clinicians in determining the appropriate timing for discontinuing RRT in ICU settings.

## Introduction and background

Acute kidney injury (AKI) is the sudden decline in the ability of the kidneys to remove waste from the body. Acute kidney injury is classified within a spectrum of conditions collectively referred to as acute kidney diseases and disorders (AKDs). These conditions are characterized by a gradual decrease in kidney function or ongoing kidney dysfunction, which can cause permanent damage to kidney cells and nephrons. This damage can potentially lead to the development of chronic kidney disease (CKD) [1].

Acute kidney injury commonly arises as a complication in hospitalized or severely ill individuals. Moreover, AKI has shown a steady increase in prevalence over the last decade. Approximately 50% of patients in the intensive care unit (ICU) are predicted to have AKI [2]. Furthermore, AKI is linked to notable illness and death rates, and kidney hurt severity plays a crucial role in determining both short-term and long-term outcomes [3].

After hospital discharge, persons who have survived a severe illness and AKI face significant challenges as they slowly recover their emotional, physical, and mental health. The long-term consequences of AKI include persistent kidney damage and a sharp susceptibility to developing CKD and ultimately renal failure [4]. Even a mild episode of AKI carries a notable risk of sustained decline in kidney function at the 90-day mark [5].

Patients who require dialysis due to AKI upon discharge represent a particularly vulnerable population [6]. In addition to managing various comorbidities, these individuals also display extra difficulties in experiencing renal replacement therapy (RRT) in outpatient dialysis centers that predominantly serve patients with end-stage renal failure. These settings may not be adequately equipped to support patients with AKI who have a favorable likelihood of functional recovery [7].

Accordingly, the use of RRT has become a crucial component in the critical care treatment of patients suffering from severe acute kidney damage. It has a crucial function in rectifying metabolic disruptions, managing excessive fluid accumulation, and averting potentially fatal consequences linked to these disorders. All physicians participating in the management of critically sick patients must have a basic comprehension of renal replacement therapy [8, 9]. Renal replacement therapy encompasses two primary categories: intermittent and continuous methods. Intermittent hemodialysis and peritoneal dialysis fall under the intermittent methods. Continuous venovenous hemodialysis, continuous venovenous hemofiltration, and continuous venovenous hemodiafiltration are all examples of continuous renal replacement therapy. Furthermore, hybrid techniques, like sustained low-efficiency dialysis, aim to benefit from both categories [8].

The primary goal of all RRT techniques is to remove unwanted water and solutes from the body by utilizing a semipermeable membrane, employing either convection, diffusion, or both processes. Diffusion is the process by which solutes move across a semipermeable membrane due to a concentration gradient. Convection is the process of solutes and solvents moving together in large quantities across a semipermeable membrane. This movement is mostly determined by transmembrane tension and membrane characteristics, rather than the concentration of solutes [8, 9].

Identifying reliable indicators for predicting the satisfactory recovery of renal function after AKI and the weaning stage has received minimal attention in the current literature, although it is crucial to avoid reinitiation of RRT. This lack of focus has limited the exploration of potential indicators, such as urine output, urine chemistry, and creatinine clearance, factors that could be valuable for predicting renal function recovery [10].

Regarding the 2012 Kidney Disease: Improving Global Outcomes (KDIGO) Clinical Practice Guidelines for AKI, RRT would cease when core renal function adequately improves or when RRT is not aligned with the other objectives. Despite the increasing number of studies in this area, there is no agreement on the detected time for discontinuing RRT, leaving this aspect of AKI management still open to debate [11].

However, there is a notable amount of inconsistency in how renal RRT is started and stopped in clinical practice, with various rationales and time factors taken into account. Several clinical and biochemical indicators, including uremic symptoms, metabolic abnormalities, and fluid balance, have been used to predict initiation and weaning of RRT. However, at present, there is no standardized procedure to guide clinicians in determining the most advantageous timing for commencing or ending RRT in order to maximize patient results [12]. Furthermore, the existing evidence is constrained by significant limitations, including retrospective and post hoc secondary designs of cohort trials, limited sample magnitudes, variations in the severity of disease and features of the trial sample, discrepancies in threshold values for predictive markers, and conflicting findings for certain markers [10]. Several studies have suggested that serum creatinine, urine output, and creatinine clearance calculated from these measurements could serve as potential indicators for discontinuing RRT. However, there is ongoing debate regarding the appropriate cut-off values for these indicators. Additionally, many of these searches have determined effective cessation of RRT as keeping liberation from the therapy for either seven or 30 days. It is crucial to consider the long-term prognosis in these cases [11, 13].

Determining the ideal time for weaning acute RRT is a complex process that involves considering multiple clinical variables and measures of renal function. Moreover, the choice to cease RRT should be made individually in ICU patients. There is a significant demand for large-scale multicenter randomized studies to address the crucial query of whether unsuitable cessation of RRT with patients with acute kidney injury requiring dialysis (AKI-D) in the ICU has an impact on patient outcomes.

Future research should focus on incorporating new indicators of kidney injury and repair as well as developing real-time methods for measuring glomerular filtration rates. Accordingly, this study aimed to investigate the predictors of RRT discontinuation. Adult patients explored a daily evaluation of urine output, serum creatinine (SCr), urinary creatinine (UCr), and urinary urea (UUr) to determine the restoration of kidney function after continuous kidney replacement therapy (CKRT). Thus, developed policymaking for the termination of RRT and the follow-up for a long-standing viewpoint.

## Review

Methods

Search Strategy

In this review, we adhered to the Preferred Reporting Items for Systematic Reviews and Meta-Analyses (PRISMA) guidelines [12] for transparent reporting. To investigate predictive factors associated with the discontinuation of RRT in adults, we systematically searched the following databases: PubMed (Medical Literature Analysis and Retrieval System Online (MEDLINE)), Excerpta Medica database (Embase), and the Cochrane Library.

The search was conducted in the databases from the beginning until December 1, 2022. Only English language literature was included. Studies were identified using Medical Subject Headings (MeSH) terms including "Termination " , "Weaning" , "Stopping" , "Discontinuation" , "Terminated" , "Stopped", "Discontinued" , "Weaned" , "Continuous Renal Replacement Therapy" , "Acute Kidney Injury". The study protocol for this research was registered through the International Prospective Register of Systematic Reviews (PROSPERO CRD 42023394020).

Study Selection

The search strategy was independently tested by two authors. Full-text articles were screened based on the inclusion and exclusion criteria for further data extraction and quality assessment. Any disagreement was discussed and agreed upon with a third reviewer.

The search approach yielded a set of documents, which were imported into the EndNote program (Clarivate, London, UK). Subsequently, duplicate articles, titles, and abstracts were removed. Two authors independently evaluated the papers to determine their eligibility. The selection process was separated into two sections. The titles and abstracts of all acknowledged publications were analyzed. Papers that satisfied the inclusion criteria or had uncertainties were selected for additional evaluation. Subsequently, two other authors evaluated the whole versions of the texts. Ultimately, the first author successfully resolved all conflicts [14].

Inclusion and Exclusion Criteria

This study encompassed observational research involving adult patients receiving renal replacement therapy (including ischemic heart disease (IHD), sustained low-efficiency dialysis (SLED), and CRRT) while supported in an ICU setting.

The studies included in this analysis investigated various parameters associated with weaning or discontinuation from continuous CRRT in severe AKI patients. These parameters included clinical, physiological, and biochemical factors, with no restrictions based on sex or geographic location.

According to the KDIGO guideline, termination of CKRT is recommended when it is no longer required after achieving sufficient renal recovery. Additionally, CKRT can be ceased if it no longer aligns with the overall treatment plan goals [11].

The utilization of diuretics to improve kidney function recovery or reduce the period or occurrence of kidney replacement therapy (KRRT) is not encouraged. There is a lack of well-established predictors to determine the appropriate timing for discontinuing CKRT or to facilitate discussions with families regarding successful discontinuation.

Moreover, various parameters, such as urine output, SCr, UCr, and urinary urea (UUr), were compared between two groups: the success group (patients who remained free from RRT within an exact time next to early termination of RRT) and the failure group (patients who required RRT within a specific time after initial discontinuation of RRT).

The evaluated outcomes included urine output, serum urea and creatinine, creatinine clearance, urinary urea, nonrenal Sepsis-related Organ Failure Assessment (SOFA) score, mean arterial pressure (MAP), neutrophil gelatinase-associated lipocalin (NGAL) levels, use of mechanical ventilation, use of vasopressors, duration of continuous CRRT, use of diuretics, length of stay in the ICU, ICU mortality rate, length of stay in the hospital, and hospital mortality rate.

However, articles with a response rate < 20%, with language other than English, or those that did not meet the previous criteria were excluded.

Data Extraction

Two independent researchers conducted eligibility evaluations, and any disagreements were resolved through consensus. In cases where a consensus could not be reached, a third researcher was involved to resolve any inconsistencies.

**Figure 1:**
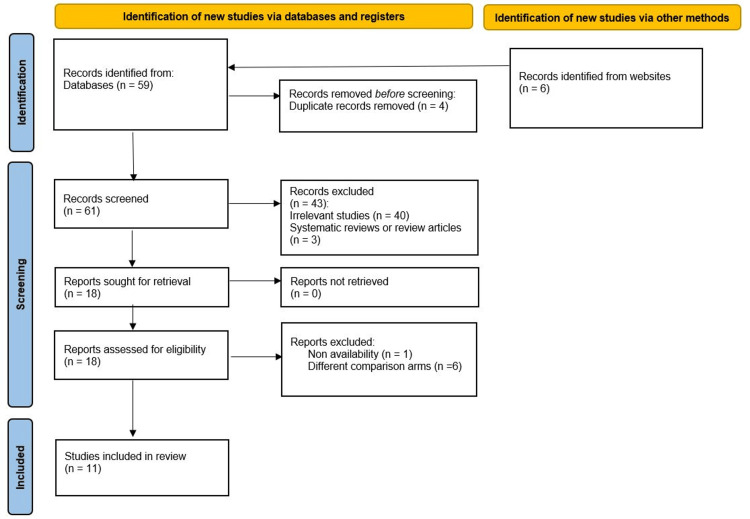
A PRISMA flow chart of the included studies PRISMA: Preferred Reporting Items for Systematic reviews and Meta-Analyses

EndNote was used to eliminate duplications. After the screening, only studies that met the inclusion criteria were included. Irrelevant topics were excluded using titles and abstracts. The full-text screening was conducted on the matched articles only. Search results are summarized using a PRISMA flow chart (Figure 1).

A total of 59 records from electronic databases (PubMed, Embase, and the Cochrane Library) were identified up to December 2022, and six additional studies were obtained through manual searching.

Among the identified records, 18 potential studies were assessed in full text, including 11 studies in our review. A total of 54 studies were excluded. There was no evidence of publication bias for the outcomes of urinary output and serum creatinine, as indicated by a p-value greater than 0.05 (Figure 1).

Quality Assessment

The quality of included studies was independently assessed using the Newcastle-Ottawa Scale (NOS) [15] for observational studies by two authors. This tool evaluates the quality of observational studies based on three essential domains: subject selection, the equivalence of individuals regarding demographics, critical potential confounders, and the ascertainment of the predetermined outcome.

The final collective score that each study could obtain ranged from 0 to nine, where a score ≥ seven was classified as a good-quality trial. Moderate quality was assigned to studies with a score of four to six, and poor quality to those with a score of < three.

Statistical Analysis

The Review Manager V.5.3.4 (RevMan (computer program), version 5.4.1, The Cochrane Collaboration, 2020) was used to calculate the overall difference in marker levels between the success and failure groups, employing the Mantel-Haenszel random effects model. Statistical heterogeneity across studies was assessed using Cochrane's Q test and the I^2^ test. The duration of urine collection in all studies was standardized to 24 hours. Additionally, mean and standard deviation were used to express all the data consistently.

The fixed-effects model [15] was utilized to calculate the pooled odds ratios (ORs) if the level of heterogeneity was deemed satisfactory (I² ≤ 50% and the Q statistic’s p-value was more than 0.1). Conversely, a random-effects model was employed if the heterogeneity exceeded these thresholds (I²≥50% or the p-value of the Q statistic was ≤ 0.1). Moreover, subgroup and sensitivity analyses were performed to identify the sources of heterogeneity. Meta-analysis and funnel plotting were conducted using Revman 5.4.1, while R version 1.1.463 (The R Core Team, R Foundation for Statistical Computing, Vienna, Austria) was used to evaluate publication bias and conduct Egger's test when an adequate number of studies were included.

Results

Study Characteristics

Our search included 11 final trials [16-26]. The comprehensive characteristics of the articles included in this study are presented in Table 1. The sample size of the studies ranged from 54 to 529, and they were predominantly observational in nature.

**Table 1:** General characteristics of the included studies * According to the duration of CRRT RRT: renal replacement therapy; CRRT: continuous renal replacement therapy

First author and year	Country	Study design	Age (mean)		Sex (male)		Type of intervention		Total number of participants	*Definition of success	
			Exp	Control	Exp	Control	Exp	Control			
											Uchino et al., 2009 [16]	Japan, Australia, Singapore, Germany, Belgium, Netherlands, Brazil, Canada, Birmingham, Italy	Post hoc analysis of a prospective observational study	62.66 (19.27)	62 (16.3)	218	146	Success	Repeated RRT	1006 (Final 529)	Unknown	
Jeon et al., 2018 [17]	Korea	Retrospective cohort study	62.1 (15.2)	61.9 (14.8)	330	222	CRRT discontinuation group	CRRT re-initiation group	2225 (Final 866)	< 7 days	
Jeon et al., 2018 [17]	Korea	Retrospective cohort study	61.0 (14.9)	61.9 (14.8)	197	222	HD initiation group	CRRT re-initiation group	2225 (Final 659)	< 7 days	
Heise et al., 2012 [18]	Germany	Retrospective single-center observational study	70.6 (8.89)	71 (12.6)	71	76	Recovery	Repeated CRRT	605 (Final 225)	Unknown	
Katayama et al., 2016 [19]	Japan	Multicentre, retrospective observational study	68 (14)	68.33 (14.1)	77	60	Success	Failure	343 (Final 254)	Unknown	
Katayama et al., 2016 [19]	Japan	Multicentre, retrospective observational study	68 (14)	68.33 (14.1)	77	60	Success	Failure	343 (Final 254)	< 7 days	
Viallet et al., 2016 [20]	France	Prospective observational study	62.8(11.1)	66.5(14.08)	20	16	The success of RRT weaning	Failure of RRT weaning	438 (Final 54)	< 14 days	
Viallet et al., 2016 [20]	France	Prospective observational study	62.8 (11.1)	66.5 (14.08)	20	16	The success of CRRT weaning	Failure of CRRT weaning	438 (Final 54)	Unknown	
Viallet et al., 2016 [20]	France	Prospective observational study	62.8 (11.1)	66.5 (14.08)	20	16	The success of CRRT weaning	Failure of CRRT weaning	438 (Final 54)	Unknown	
Yoshida et al., 2019 [21]	Japan	A retrospective single-center cohort study	64 (17.8)	64.66 (14.8)	25	12	Discontinued CRRT	Continued CRRT	68 (Final 52)	< 7 days	
Yoshida et al., 2019 [21]	Japan	A retrospective single-center cohort study	64 (17.8)	64.66 (14.8)	25	12	Discontinued CRRT	Continued CRRT	68 (Final 52)	Unknown	
Liu et al., 2021 [22]	USA, China	A single-center, retrospective cohort study	63.35 (14.2)	62.65 (15.6)	112	422	Successful	Unsuccessful	1135 (Final 909)	< 7 days	
Baeg et al., 2021 [23]	Korea		63.8	61.2	366	358	Successful discontinuous CRRT group	Failure discontinuous CRRT group	1158	Unknown	
Stads et al., 2019 [24]	Netherlands	A prospective multicentre study	62	64	38	17	Successful stop CRRT group	Unsuccessful stop CRRT group	92	< 7 days	
Chen et al., 2019 [25]	China	A prospective observational study	50.2	52.5	46	22	Discontinuation of CRRT was successful	Discontinuation of CRRT failure	110	Unknown	
Thomsen et al., 2020 [26]	Denmark	A prospective observational study	72.9	68.9	16	11	Discontinuation of CRRT was successful	Discontinuation of CRRT and re-initiation	42	Unknown	

The assessment of weaning from RRT was conducted by physicians based on their clinical expertise. Successful weaning was defined using different criteria and time frames, with the discontinuation of dialysis ranging from seven to 30 days.

Risk of Bias in Studies

Among the 11 assessed cohort studies, nine were of good quality (NOS scores ≥7), and two were of moderate quality (NOS scores = 6),as shown in Table 1**.**

Clinical Outcomes

Prediction of RRT cessation by urine output: A total of 10 studies investigated the anticipatory role of urine output. In the trial conducted by Viallet et al. [20], it was noted that urine output alone was insufficient to forecast dialysis success. However, other studies suggested that urine output could serve as a predictor of dialysis success.

**Figure 2:**
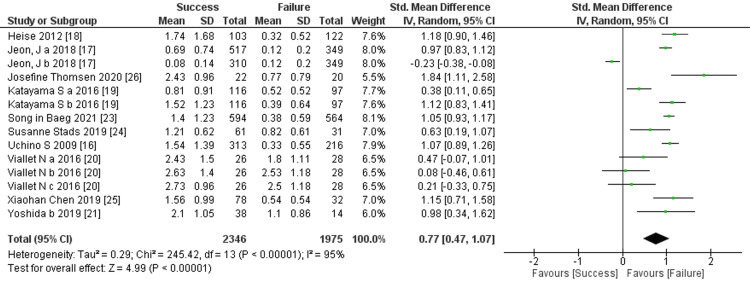
Forest plot of comparison: urinary output (L/day) between success and failure groups. Sources: [16-21, 24-26]

Our analysis revealed a significant correlation between urine output and dialysis weaning success. Patients who successfully weaned had notably higher urine output compared to those who failed to wean, indicating its predictive value (10 trials, 4,321 participants; std mean difference: 0.77; 95% CI: 0.47-1.07, p < 0.00001; heterogeneity: p < 0.00001, I^2^ = 95%) (Figure 2).

Prediction of RRT cessation by renal function: Serum creatinine was utilized in 10 studies to predict the termination of RRT. The comprehensive examination revealed a notable difference in the average serum creatinine levels between the success and failure groups.

**Figure 3:**
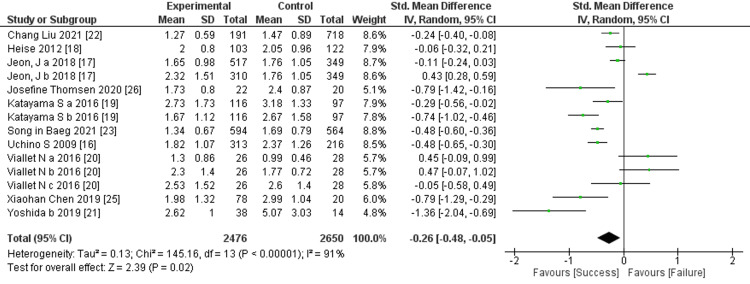
Forest plot of comparison: Serum creatinine (mg/dl) between the success and failure groups. Sources: [16-23, 25, 26]

Specifically, the failure group had significantly higher average serum creatinine levels compared to the success group, indicating a significant difference between the two groups (10 trials, 5,126 participants; std mean difference: −0.26; 95% CI: −0.48 to −0.05, p = 0.02; heterogeneity: p < 0.00001, I² = 91%) (Figure 3).

**Figure 4:**
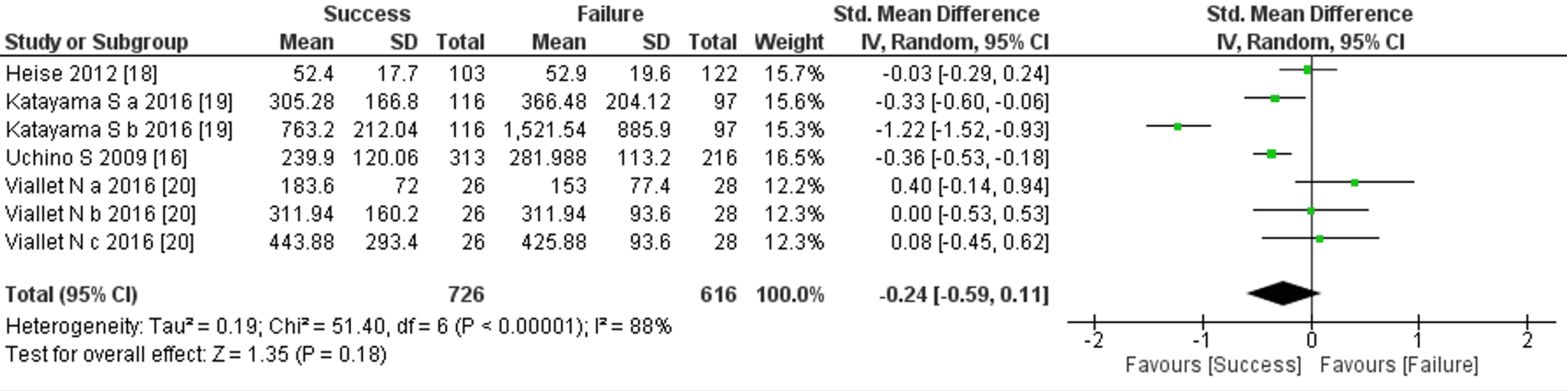
Forest plot of comparison: serum urea (mg/dl) between the success and failure groups. Sources: [16, 18-20]

However, among the four studies examining serum urea as a predictor for discontinuation of RRT, two concluded that serum urea levels did not significantly differ between the success and failure groups. The overall findings from the comprehensive examination also indicated no significant difference in serum urea levels between the two groups (four trials, 1342 participants; std mean difference: −0.24; 95% CI: −0.59 to 0.11, p = 0.18; heterogeneity: p < 0.00001, I² = 88%) (Figure 4).

**Figure 5:**
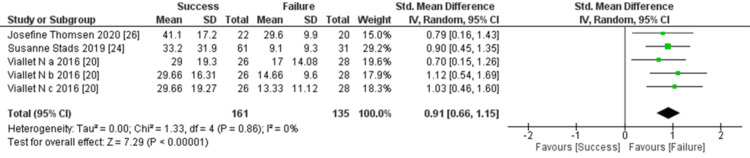
Forest plot of comparison: Creatinine clearance (ml/min) between the success and failure groups. Source: [20, 24, 26]

Besides, among the three studies investigating creatinine clearance as a predictor for discontinuation of RRT, a significant difference was observed between the two groups. The comprehensive analysis consistently showed significant differences in creatinine clearance levels between the two groups (three trials, 296 participants; Std mean difference: 0.91; 95% CI: 0.66 to 1.15, p < 0.00001; heterogeneity: p = 0.86, I² = 0%) (Figure 5).

**Figure 6:**
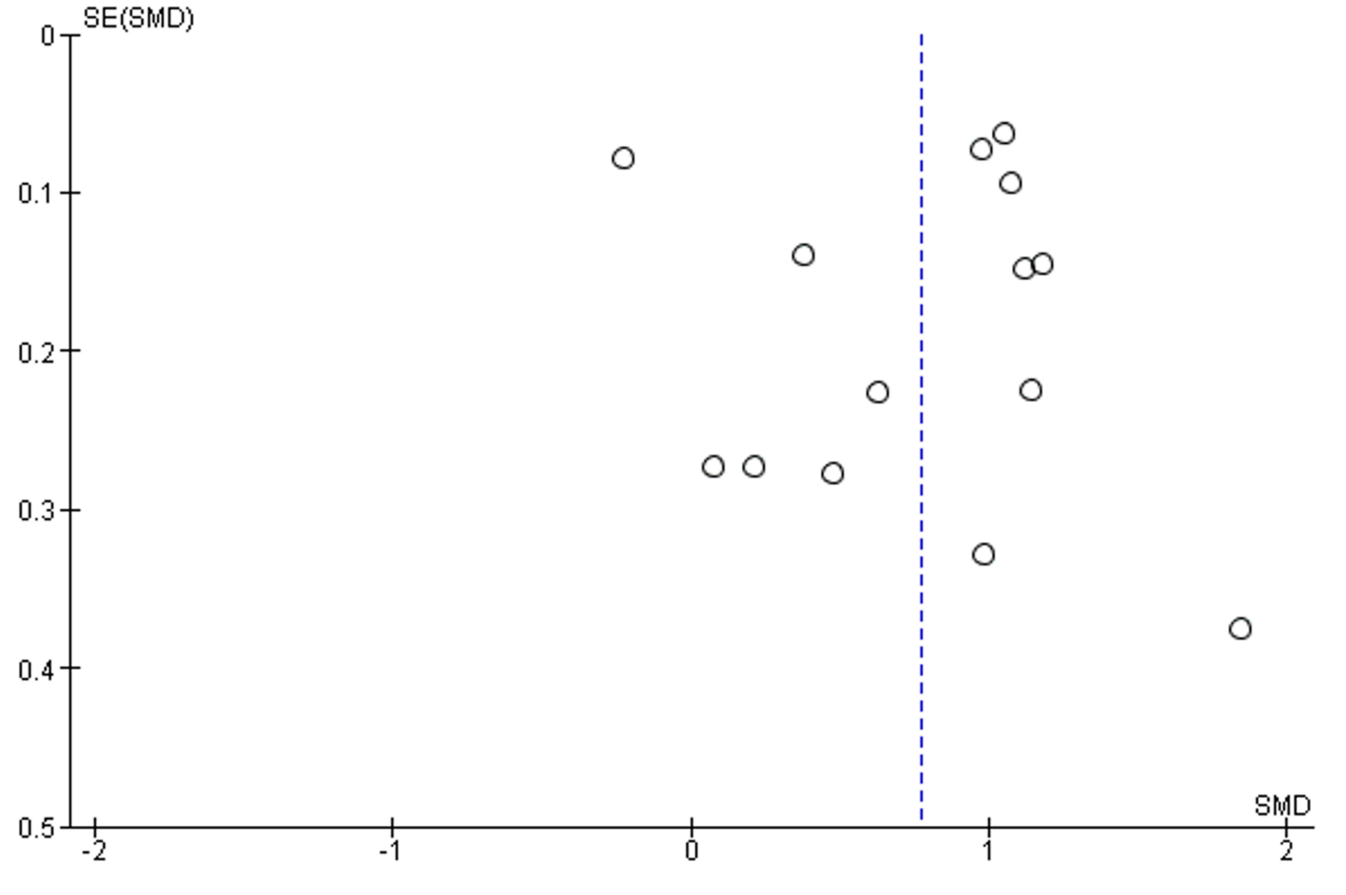
Funnel plot of urinary output (L/day) between success and failure groups SMD: standard mean difference; SE: standard error

**Figure 7:**
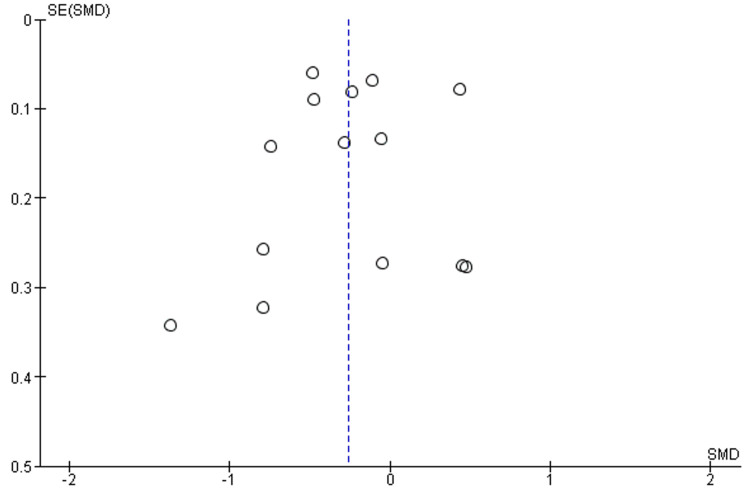
Funnel plot serum creatinine (mg/dl) between success and failure groups SMD: standard mean difference; SE: standard error

The funnel plot figures displayed the comparison between the two groups in terms of urine output and serum creatinine (Figures 6, 7).

**Figure 8:**
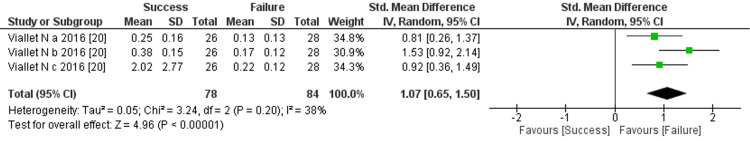
Forest plot of comparison: urinary creatinine (mg/dl) between the success and failure groups. Source: [20]

Prediction of RRT cessation by urinary biochemical marker: The comprehensive analysis revealed a significant disparity in urinary creatinine levels between the studied groups (one trial, three subgroups (urinary creatinine at D0, D1, D2), 162 contributors; std mean difference: 1.07; 95% CI 0.65 to 1.5; p < 0.00001; heterogeneity: p = 0.2, I^2^ = 38%) (Figure 8)**.**

**Figure 9:**
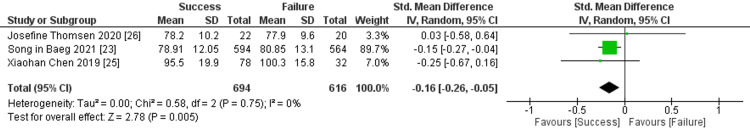
Forest plot of comparison: mean arterial pressure (MAP; mmHg) between the success and failure groups. Source: [23, 25, 26]

Prediction of RRT cessation by hemodynamic stability: Three trials addressed mean arterial pressure as a variable: two studies reported no statistical difference between the two groups, while one study identified a significant difference (three trials, 1,310 participants; std mean difference: -0.16; 95% CI: −0.26 to -0.05, p = 0.005; heterogeneity: p = 0.75, I^2^ = 0%) (Figure 9).

**Figure 10:**
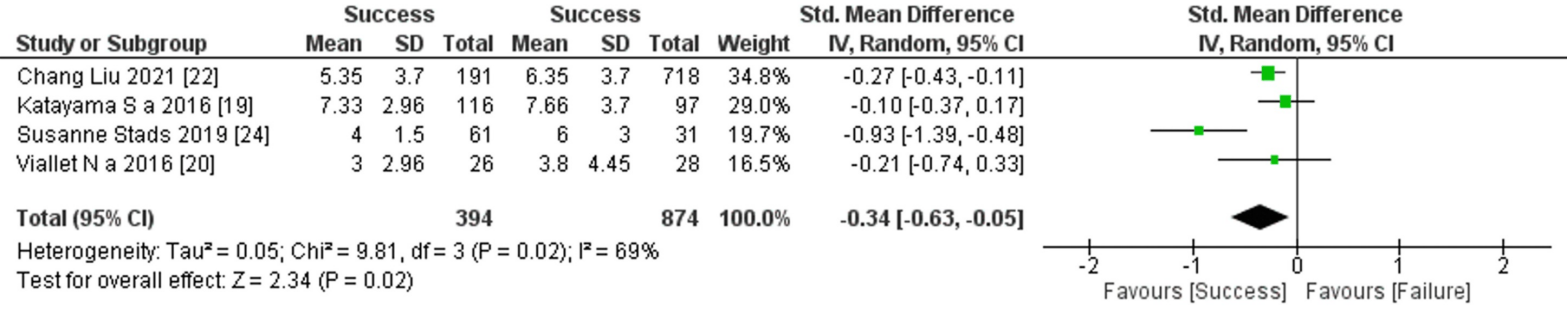
Forest plot of comparison: nonrenal Sepsis-related Organ Failure Assessment (SOFA) score between the success and failure groups. Source: [19, 20, 22, 24]

Prediction of RRT cessation by nonrenal SOFA score: Four studies mentioned non-renal SOFA scores, and the comprehensive examination of these scores detected a significant difference between the two groups (four studies, 1,268 contributors; std mean difference: −0.34; 95% CI −0.63 to -0.05, p = 0.02; heterogeneity: p = 0.02, I^2^ =69%) (Figure 10).

**Figure 11:**
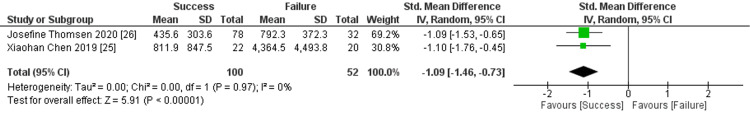
Forest plot of comparison: neutrophil gelatinase-associated lipocalin (NGAL) between the success and failure groups. Source: [25, 26]

Prediction of RRT cessation by NGAL: Two studies mentioned NGAL, and a comprehensive analysis of NGAL showed a significant difference between the two groups (two studies, 152 contributors; std mean difference: −1.09; 95% CI −1.46 to -0.73, p <0.00001; heterogeneity: p = 0.97, I2 =0%) (Figure 11).

**Figure 12:**
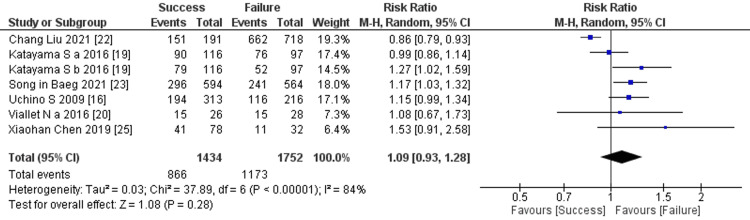
Forest plot of comparison: use of mechanical ventilation between the success and failure groups. Source: [16, 19, 20, 22, 23, 25]

Prediction of RRT cessation by use of mechanical ventilation: Six studies mentioned the use of mechanical ventilation, and the comprehensive analysis of the outcome detected no significant difference between the two groups (six studies, 3,186 participants; risk ratio: 1.09; 95% CI 0.93 to 1.28, p = 0.28; heterogeneity: p < 0.00001, I^2^ = 84%) (Figure 12).

**Figure 13:**
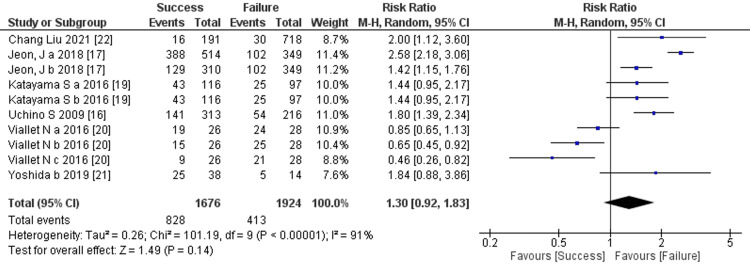
Forest plot of comparison: use of diuretics between the success and failure groups. Source: [16, 17, 19-22]

Prediction of RRT cessation by use of diuretics: Six studies mentioned the use of diuretics, and the comprehensive analysis of the outcome detected no significant difference between the two groups (six trials, 3,600 participants; risk ratio: 1.3; 95% CI 0.92 to 1.83, p = 0.14; heterogeneity: p < 0.00001, I2 = 91%) (Figure 13).

**Figure 14:**
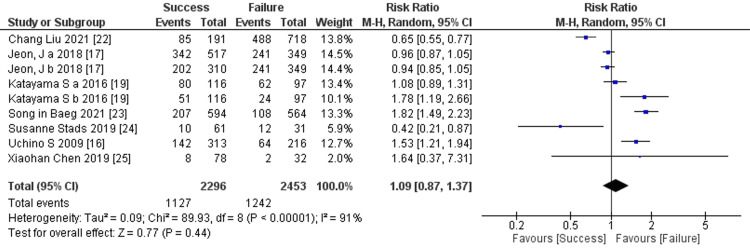
Forest plot of comparison: use of vasopressors between the success and failure groups. Source: [16, 17, 19, 22-25]

Prediction of RRT cessation by use of vasopressors: Seven studies mentioned the use of vasopressors, and the comprehensive analysis of the outcome showed no significant difference between the two groups (seven trials, 2,369 participants; risk ratio: 1.09; 95% CI 0.87 to 1.37, p = 0.77; heterogeneity: p < 0.00001, I² = 91%) (Figure 14).

**Figure 15:**
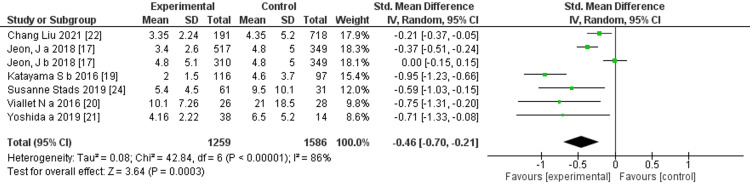
Forest plot of comparison: continuous renal replacement therapy (CRRT) duration between the success and failure groups. Source: [17, 19-22, 24]

Prediction of RRT cessation by CRRT duration days: Six studies mentioned the CRRT duration days, and the comprehensive analysis of the outcome showed a significant difference between the two groups (six trials, 2,369 participants; std mean difference: -0.46; 95% CI -0.7 to -0.21, p = 0.0003; heterogeneity: p <0.00001, I^2^ =86%) (Figure 15).

**Figure 16:**
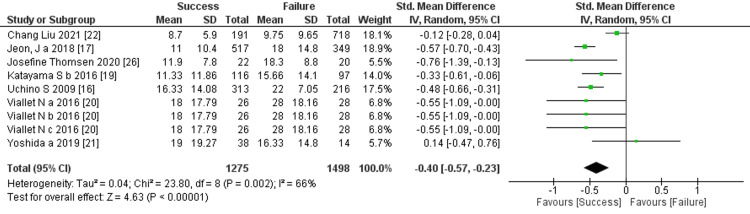
Forest plot of comparison: length of ICU stay between the success and failure groups. Source: [16, 17, 19-22, 26]

Prediction of RRT cessation by the length of ICU stay: Seven studies mentioned the length of ICU stay, and the comprehensive analysis of the outcome showed a significant difference between the two groups (seven trials, 2,773 participants; std mean difference: -0.4; 95% CI -0.57 to -0.23, p < 0.00001; heterogeneity: p < 0.002, I^2^ = 66%) (Figure 16).

**Figure 17:**
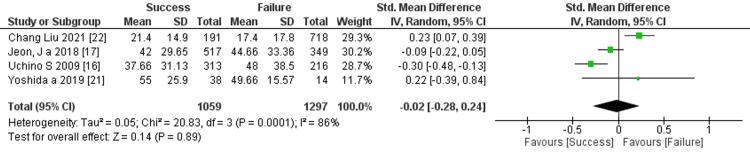
Forest plot of comparison: length of hospital stay between the success and failure groups. Source: [16, 17, 21, 22]

Prediction of RRT cessation by the length of hospital stay: Four studies mentioned the length of hospital stay, and the comprehensive analysis of the outcome showed no significant difference between the two groups (four trials, 2,356 participants; std mean difference: -0.02; 95% CI -0.28 to 0.24, p =0.89; heterogeneity: p =0.0001, I^2^ =86%) (Figure 17).

**Figure 18:**
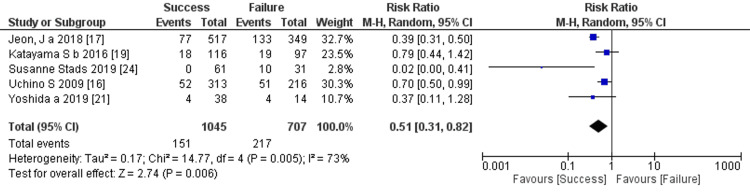
Forest plot of comparison: ICU mortality between the success and failure groups. Source: [16, 17, 19, 21, 24]

Prediction of RRT cessation by ICU mortality: Five studies mentioned ICU mortality, and the comprehensive analysis of the outcome showed a significant difference between the two groups (five trials, 1,752 participants; risk ratio: 0.51; 95% CI 0.31 to 0.82, p = 0.006; heterogeneity: p = 0.005, I² = 73%) (Figure 18).

**Figure 19:**
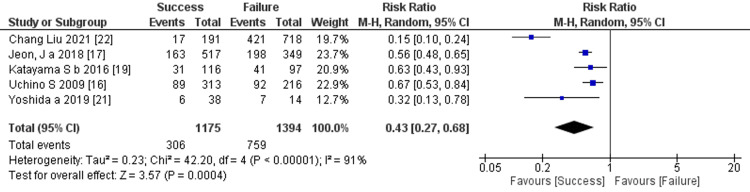
Forest plot of comparison: hospital mortality between the success and failure groups. Source: [16, 17, 19, 21, 22]

Prediction of RRT cessation by hospital mortality: Five studies mentioned hospital mortality, and the comprehensive analysis of the outcome showed a significant difference between the two groups (five trials, 2569 participants; risk ratio: 0.43; 95% CI 0.27 to 0.68, p =0.0004; heterogeneity: p <0.00001, I^2^ =91%) (Figure 19).

Discussion

This systematic review and meta-analysis evaluated 11 included articles for possible predictors of CRRT discontinuation. The 11 studies showed a significant difference between the two review groups (success and failure) regarding the urinary output cut-off level (p <0.00001). This could be due to the variability of the identified normal daily UO levels in the included studies, which ranged from 0.08 to 2.733 L/day.

Furthermore, the comprehensive analysis results indicated significant differences in creatinine clearance, CRRT duration, and nonrenal SOFA scores between the two groups (p < 0.00001, p = 0.0003, and p = 0.02, respectively). Our findings suggest that the serum creatinine level upon discontinuing CRRT can be a predictive factor for successful termination. A previous meta-analysis reached a similar conclusion [27]. In fact, cumulative urine output 24 hours before the cessation of CRRT (cut-off 210 ml) showed superiority with a predictive value of 93% when combined with urinary neutrophil gelatinase-associated lipocalin (uNGAL) [16, 17, 19, 22].

The last meta-analysis [28] demonstrated that CKD, duration of CRRT, and urine output (with an increase of 100 mL/day) at the discontinuation of CRRT were significant predictors of short-term weaning success. However, the strength of these associations was not firmly established for the use of vasopressors or inotropes at the initiation or cessation of CRRT, the use of diuretics upon CRRT cessation, or serum creatinine levels at CRRT discontinuation.

It is important to note that this recent study faced notable heterogeneity across the included studies, including variations in the definition of weaning success, substantial differences in study design, and a limited number of studies evaluating the SOFA score. Furthermore, the inclusion of cross-sectional studies did not allow for the verification of causal associations.

Our review differs from the previous systematic reviews in several ways. It found that six of the 11 included studies used diuretics, with no significant difference between success and failure groups. According to Jeon’s study [17], successful discontinuation of CRRT was associated with aggressive administration of diuretics the day before discontinuation. In addition, elevated urinary output with increased serum creatinine levels was detected after CRRT was discontinued in the diuretic group. However, a significant predictor was urinary output higher than 0.5 mL/kg/h despite the diuretic use six hours before CRRT discontinuation [29].

Furthermore, uNGAL levels were correlated with acute kidney worsening in unstable nephropathies [30, 31], with possible involvement in the pathogenesis of CRD, including polycystic kidney disease and glomerulonephritis [30]. This finding aligns with the pooled results of both included studies, which showed a significant difference between the two groups (heterogeneity: p = 0.97, I² = 0%) [25, 26].

In this pooled analysis, a shorter duration of CRRT, lower ICU mortality, and ICU length of stay were associated with a significant difference between the two groups in favor of the failure group (all with significant heterogeneity, P< 0.05). Other studies revealed similar results as strong predictors for the successful weaning of CRRT [10, 29, 32, 33].

Moreover, as prolonged ICU and hospital stays may indicate respiratory failure in patients with AKI, mechanical ventilation was detected as an indicator of failure to wean from CRRT [34, 35]. However, the mean difference between success and failure groups in our pooled analysis showed no significant association with mechanical ventilation use.

Furthermore, AKI is usually associated with hemodynamic instability, which requires establishing definite therapeutic hemodynamic goals and maintaining circulatory stability during CRRT [36]. Hemodynamic instability was found to be associated with early mortality and an increase in SOFA score [37, 38], while SOFA score could not predict successful weaning in the studies by Tian et al. and Li et al., which agree with the results of our pooled analysis [32, 39].

The use of vasopressors before the initiation of CRRT was linked to higher mortality rates [40]. Furthermore, vasopressors showed a higher predictive level for increased mortality among AKI patients on CRRT [39]. Conversely, the interval from vasopressor initiation to CRRT initiation (Tvaso-CRRT) was found to be significantly correlated with higher survival compared to non-survival patients (84.3% vs. 58.5%, p < 0.001) [41]. Unfortunately, this pooled analysis could not detect any significant difference between success and failure groups regarding vasopressor use. 

This study stands out in its comprehensive identification and synthesis of an extensive range of predictive variables related to physiological and biochemical parameters contributing to effective termination from CRRT, a dimension lacking in previous systematic reviews. Notably, we enhanced the predictive capacity of urine output by incorporating patient-specific variables (e.g., CRRT duration), biomarkers (e.g., NGAL), and clinical severity scores (e.g., SOFA score). By integrating these diverse factors, we improved the accuracy of predicting successful weaning from CRRT.

Moreover, shorter duration of CRRT, lower ICU mortality, and ICU length of stay were associated with a significant difference favoring the failure group over the success group. Finally, we strictly adhered to the Cochrane Library and PRISMA guidelines.

There are some limitations to be acknowledged. Firstly, although all the studies included in our analysis were cohort studies, there was notable heterogeneity among them, likely due to differences in how successful weaning from CRRT was defined across the included studies. Furthermore, the decision to discontinue CRRT was largely based on physicians' discretion rather than standardized criteria in the studies we examined. Lastly, our study did not specifically analyze the impact of comorbidities.

Additionally, while our literature search was comprehensive from the beginning up to December 1, 2022, newer studies published in 2023 and 2024 may provide further insights. This time limitation will be important to consider in future updates. Although we believe the current timeframe aligns with our PROSPERO-registered protocol, we acknowledge this as a limitation and encourage future systematic reviews to capture emerging data.

In clinical practice, effective termination of CRRT is often defined as being free from CRRT for seven to 14 days. However, it is worth noting that the effective termination of kidney replacement therapy (KRT) may hold greater importance than simply discontinuing CRRT. Future research on integrating artificial intelligence (AI) and clinical electronic data is warranted [42, 43].

## Conclusions

Our study and the analysis revealed that various variables, including urine output (with an increase of 100 mL/day), serum creatinine levels, CRRT duration, and mortality at the cessation of CRRT, serve as predictors for weaning from CRRT. These findings provide valuable insights into the factors associated with successful discontinuation of CRRT. However, limited evidence was found regarding the specific threshold for optimum urine output and serum creatinine levels to predict successful weaning of CRRT. Future controlled trials are recommended to assess the optimum threshold for each variable and to clarify these associations, thereby better guiding clinicians on when to discontinue RRT in ICU settings.
